# The effect of triple antibiotic paste as an intracanal medicament with an anti-inflammatory agent on post-operative pain of asymptomatic uniradicular necrotic teeth: a double blind randomized clinical trial

**DOI:** 10.12688/f1000research.19699.3

**Published:** 2025-12-16

**Authors:** Mohamed Omaia, Maged Negm, Yousra Nashaat, Nehal Nabil, Amal Othman

**Affiliations:** 1 Department of Endodontics, Faculty of Dentistry, October 6 university, Giza, Egypt; 2 Department of Endodontics, Faculty of Dentistry, Cairo university, Cairo, 11553, Egypt; 3 Department of Microbiology, Faculty of Applied Medical Sciences, October 6 University, Giza, Egypt

**Keywords:** TAP, diclofenac potassium, anti-inflammatory, calcium hydroxide, intracanal medication, necrotic, post-operative pain.

## Abstract

**Background:**

Flare-ups may occur after root canal treatment which consist of acute exacerbation of asymptomatic pulpal and/or periradicular pathologic condition. The causative factors of interappointment pain include mechanical, chemical or microbial irritation to the pulp or periradicular tissues. The potential role of microorganisms in interappointment pain is why the success of endodontic treatment depends on complete eradication of microorganisms capable of causing an intraradicular or extraradicular infection. This can be achieved by mechanical cleaning and shaping, in conjunction with irrigation and antibacterial agents. The aim of this study was to assess the ability of triple antibiotic paste with the anti-inflammatory drug diclofenac potassium versus calcium hydroxide used as an intra-canal medication in reducing post-operative pain.

**Methods:**

84 patients with asymptomatic uniradicular necrotic teeth were randomly assigned into two groups according to the intra-canal medication used: calcium hydroxide group (CH) and triple antibiotic paste with diclofenac potassium group (TAPC). In the first treatment session, intracoronal cavity preparation was performed after rubber dam isolation followed by chemo-mechanical preparation using rotary Protaper Universal files with saline irrigation followed by intra-canal medication placement then postoperative pain was assessed at 24, 48 and 72 hours postoperatively using Visual Analogue Scale (VAS). In the second treatment session, intracanal medications were removed by irrigation using saline followed by obturation.

**Results:**

Both intracanal medicaments resulted in a statistically significant decrease in mean pain value from 24 to 48 and 72 hours postoperatively. While when comparing both groups, TAPC intracanal medication showed less post-operative pain compared to that of the CH group at 24, 48 and 72 hours with a statistically significant difference at 48 hours only.

**Conclusion:**

Both intracanal medicaments were efficient in reducing post-operative pain in asymptomatic uniradicular necrotic teeth.

**Trial registration:**

Clinicaltrial.gov NCT02907489, 20/09/2016.

## Introduction

The severity and incidence of post-operative pain are usually associated with specific dental treatments; the highest of which is root canal therapy ^1^.

Post-operative pain is a common finding after endodontic treatment, its incidence ranges from 3% to 58%. It may be due to microbial, mechanical or chemical injury to the periapical tissues ^2, 3^. It is the main reason why patients seek endodontic treatment, therefore it must be accompanied by efficient relief of pain to be considered successful by both patients and clinicians ^4^.

 Indeed, acute periradicular inflammation such as acute periodontitis or acute periradicular abscess secondary to intracanal procedure is one of the causes of postoperative pain ^5, 6^. Infected debris from the root canal system may induce an acute inflammatory response of the tissues in response to the applied irritants ^7^.

 Microorganisms are the primary cause for pulp and periapical diseases. The failure to eliminate them and their by-products may result in persistent irritation and impaired healing ^8^.

Pain is a subjective experience and difficult to quantify and standardize. Pain is influenced by many factors e.g. personality, behavior, physical and psychological factors; making it a challenge to measure. Several scales and methods have been used for the assessment of pain after endodontic therapy. Among them, numerical, verbal and visual analogue scales are used in most clinical studies ^9^.

A visual analogue scale was used to assess postoperative pain. This is a valid and reliable method which has been widely used in endodontic literature ^10, 11^.

 Post-endodontic pain, particularly after initial root canal treatment, should ideally be eliminated by the therapy; however, analgesics and/or anti-inflammatory drugs are frequently required to reduce post-operative pain ^9^.

Endodontic therapy is directed toward one specific aim: to cure or prevent periradicular periodontitis. Our challenge as endodontists is to implement methods to eliminate these microorganisms during and after root canal treatment.

It has been suggested that calcium hydroxide has pain preventive properties because of its antimicrobial and tissue altering effects ^12^. Furtheremore studies have shown that calcium hydroxide has the ability to dissolve necrotic tissues alone or may increase the effect of other solvents. This suggests that calcium hydroxide would be an effective agent in cleaning and disinfecting infected root canals ^13^. 

Systemic antibiotics appear to be clinically effective as an adjunct in certain surgical and nonsurgical endodontic procedures. Their administration is not without potential risk of adverse systemic effects, such as toxicity, allergic reactions and the development of resistant strains of microbes ^14^. Furthermore, the systemic administration of antibiotics depends on patient’s compliance with the dosing regimens followed by absorption through the gastro-intestinal tract and distribution via the circulatory system to bring the drug to the infected site. Hence, the infected area requires a normal blood supply which is no longer the case for teeth with necrotic pulps and for teeth without pulp tissue. Therefore, local application of antibiotics within the root canal system may be a more effective mode for delivering the drug ^15^.

 A successful endodontic treatment is therefore dependent on the initial eradication of all the bacteria, i.e. those present in the root canal as well as those already penetrated in depth ^16^. The achievement of microbicidal doses becomes critical in the endodontic environment, because in such harsh conditions bacteria may aggregate to form a biofilm or enter a stationary phase, thus acquiring a resistant phenotype. Accordingly, in endodontic therapy the local use of antibiotics allows the use of the necessary very high concentrations ^17^.

 Because root canal infections are polymicrobial, consisting of both aerobic and anaerobic bacterial species, a single antibiotic may not be effective in root canal disinfection. Therefore, a combination of antibiotics, mainly consisting of ciprofloxacin, metronidazole and minocycline, referred to as triple antibiotic paste (TAP) has been suggested for root canal disinfection ^18, 19^. The aim of the present study is to evaluate the amount of post operative pain after using triple antibiotic paste with an anti-inflammatory drug as an intracanal medicament and to compare it with calcium hydroxide in patients with asymptomatic uniradicular necrotic teeth.

The null hypothesis is that there is no significant difference in post operative pain level after using triple antibiotic paste with an anti-inflammatory agent and calcium hydroxide as an intracanal medicaments in patients with asymptomatic uniradicular necrotic teeth.

## Methods

### Study setting

The study design is a parallel double blind randomized controlled clinical trial with a 1:1 allocation ratio and a Superiority Framework. The study was conducted between March 2017 and July 2018 in the Endodontic clinic of the Faculty of Dentistry, Cairo University. The protocol of this study was approved by the Ethics Committee of the Faculty of Oral and Dental Medicine, Cairo University, Egypt (approval number 16/10/28). The nature of this study, and potential risks were explained to the patients and informed consent forms were signed before treatment sessions. This trial was registered with ClinicalTrials.gov on 20 ^th^ September 2016 ( NCT02907489).

This article was written in concordance with the CONSORT checklist 2010 (see reporting guidelines).

### Sample size

To assess TAPC versus CH regarding the postoperative pain, an independent t test was done. It was estimated that a total of 75 patients would be required for the detection of a difference between groups using a two-tailed α of 0.05 and a power of 0.80 if the absolute difference in post-operative pain is 0.35 with SD 0.65 as reported in Johns *et al*., 2014 ^20^. To compensate losses during the follow-up this number should be increased to 84 patients (10% more than the calculated). Sample size was calculated using G* power program (3.1.9.2/ 2014) (University of Düsseldorf, Düsseldorf, Germany).

### Participants

The subjects were selected from the regular attendees of endodontic clinic, Faculty of Dentistry, Cairo University. A written announcement was published in the endodontic clinic to inform the attendees regarding the trial, the patients who indicated their interest to participate in the study were checked for eligibility and signed an informed consent.

 Patients were carefully diagnosed and checked for the eligibility criteria through careful clinical examination using a diagnostic mirror and probe, percussion with the back of a diagnostic mirror and palpation with the index finger to indicate the presence of any swelling or tenderness were done. An electric pulp tester (Denjoy DY310, Henan) that was positioned at the middle third of the labial/buccal surface of the tooth was used to check pulp vitality of the affected tooth, with the adjacent and contra-lateral teeth used as a control. All data was recorded in medical history charts, dental history charts and pain scale charts (Extended data ^21^).

The pain scale (0–10 scale) consisted of a 100 mm horizontal ruler with no numbers except a 0 at the first part of the scale and a 10 in the last part of the scale. The patients were asked to mark the point that was equivalent to their pain perception. The pain levels were classified as no pain (0), mild pain (1–3), moderate pain (4–7) or severe pain (8–10) and postoperative pain was assessed at 24, 48 and 72 hours after intracanal medication placement.

 The VAS is considered to be a valid and reliable scale for the measurement of pain. This method may have some limitations in terms of objectivity considering the heterogeneity of personal character ^22^. However, previous studies argued that this method can be considered adequately reliable ^23^. Therefore, VAS was used in this study to evaluate post-operative pain.

In total, 84 participants were included in the study with maxillary or mandibular asymptomatic necrotic single rooted teeth as studies have suggested that postoperative pain is more likely to occur in multirooted teeth because in multirooted tooth other variable exist which may affect our treatment outcome ^24^. Non-vital teeth were selected because research has found that post-operative pain in teeth with non-vital pulp are more common than teeth with vital pulp due to the presence of high level of irritants and infectious agents in pulpal and periapical region ^25– 27^. Asymptomatic teeth were chosen because studies have shown that the presence of preoperative pain can significantly increase the possibility of postoperative pain ^27, 28^.

*Inclusion criteria:* Subjects aged between 18–50 years. Both male and female medically free (without any systemic diseases) and healthy subjects. Mandibular and maxillary single rooted asymptomatic non vital teeth were chosen.

*Exclusion criteria:* Teeth with acute dentoalveolar abscess, subjects having more than one tooth that require root canal treatment. Subjects that have taken analgesic, anti-inflammatory or antibiotic drugs during the 10 days prior to the start of treatment. Pregnant females. Subjects with systemic diseases such as endocrine diseases, infectious diseases or psychological disturbance. Subjects taking chronic pain medications. Teeth with periodontal disease or pulp calcification.

Patients were randomly assigned into two groups (n=42/group) according to the intra-canal medication used, using computer generated randomization ( *www.random.org*). The assistant supervisor (A.O.) generated the random sequence and assigned the participants to the intervention or control groups.

Experimental group: triple antibiotic paste + Catafast (TAPC). Control group: calcium hydroxide paste (CH).

### Outcomes

*Primary outcome:* Post-operative pain change.

*Measuring device:* Visual analogue scale.

*Measuring unit:* Ordinal.

*All categories of pain considered success.

*Secondary outcome:* Intracanal bacterial reduction of each strain of bacteria obtained from the root canals before (S1) and after instrumentation (S2) in the first treatment session. Subsequently, intra-canal medication was placed and bacterial reduction was assessed in the second session after 3 days (S3) using colony forming unit test.

*Measuring device:* Bacterial culture.

*Measuring unit:* Colony forming units per milliliter of blood agar medium.

*Secondary outcome will be published in a separate manuscript.

### Intervention

*First visit:* Preoperative intraoral periapical radiograph was taken *(Kodak intraoral periapical films speed D, Size 2, KODAK)*. Teeth were anaesthetized by local anesthesia with a non-disposable metallic aspirating syringe using 1.8 ml Mepivacaine HCl 2% - Levonordefrin 1:20000 *(Carpule Mepecaine-L, Alexandria Company for Pharmaceuticals and Chemical Industries, Egypt, #1423).* An access cavity preparation was performed using round bur size 3 and endo-Z bur for deroofing under a continuous irrigation with sterile physiologic saline *(El Nasr Pharmaceutical Chemicals CO., Abu Zaabal, Egypt)*. A rubber dam was placed. The patency of the canals was checked using size 15 K-files *(MANI, INC., Tochigi, Japan).* Working length was determined using an electronic apex locator *(Root ZX, J.Morita USA, Irvine, CA)* at the “0.5” mark then was confirmed with intraoral periapical radiograph, to be 0.5–1 mm, shorter than radiographic apex. The canals were instrumented to a size 25 K-type file *(MANI, INC., Tochigi, Japan)*. Preparation of teeth was performed with rotary ProTaper Universal instruments *(Dentsply Maillefer, TN, USA)* in an endodontic motor and reducing hand piece *(X-Smart, Dentsply Maillefer, USA)*, according to the manufacturer instructions. The canals were thoroughly irrigated using 2ml of sterile physiologic saline as an irrigant between each instrument and the next. At this stage the canals were dried and filled with intra-canal medication according to the randomly selected group (TAPC group- combination of 500 mg ciprofloxacin, 500 mg metronidazole, 55 mg minocycline and 50 mg diclofenac potassium ground and then mixed with saline to obtain creamy consistency and CH group #MB-302001) ( Table 1). A cotton pellet was placed in the pulp chamber and the access cavity was sealed with a temporary filling Orafil-G *(Prevest Denpro, Digiana, India #11002).* Postoperative pain was assessed with a visual analog scale at 24, 48 and 72 hours after the procedures.

**Table 1.** Material specification, composition and manufacturer.

Material	Specification	Composition	Manufacturer
**Metapex**	Non setting Calcium Hydroxide with Iodoform	Calcium Hydroxide, Iodoform and Silicon oil.	META BIOMED Co, Chungbuk, Korea. [270 Osongsaengmyeong1-ro, Osong-eup, Heungdeok-gu, Cheongju-si, Chungbuk, Korea / Tel :043-218-1981] *http://www.meta-biomed.com*
**Triple** **antibiotic** **paste (TAP)**	Mixture of ciprofloxacin, metronidazole and minocycline.	Using commercially available tablets of Ciprofloxacin (Ciprofloxacin 500 mg), Metronidazole (Flagyl 500 mg) and Minocycline (Minocin 50 mg). Following the removal of the enteric coating of the tablets, the contents were ground using a mortar and pestle and mixed in an equal amounts by weight (1:1:1) in a mixing pad (100 mg of each) and then will be dissolved in 100 mL of sterile water to prepare 1 mg/mL solution of TAP ^31^	Flagyl 500 mg: (Sanofi Aventis, Cairo, Egypt) 3, El Massaneh St. Zietoun, Cairo, Egypt Tel.: +202 22860000 22860060/1/2 *www.sanofi.com* Ciprofloxacin 500 mg: (Amriya pharm, Alexandria, Egypt) Alexandria-Cairo Desert Rd. Km 25, Amriya, Alexandria, Egypt. Tel.: +20 (3) 470-1001 / 470-1151 / 470-1146 *http://amriyapharm.com/* Minocin 50 mg: (Sedico, Giza, Egypt) 1st. industrial zone, 6th of October City, Giza, Egypt Tel: +202-38200575/78/90 *http://www.sedico.net*
**Catafast**	50 mg NSAIDs granules for oral solution (Diclofenac potassium)	Every sachet contains 50 mg diclofenac potassium, Potassium hydrogen carbonate, mannitol; aspartame, saccharin sodium, glyceryl dibehenate, mint flavor, anise flavor.	*NOVARTIS PHARMA S.A.E., Cairo, Egypt* *[El Sawah St. – EL Amiria, Cairo, Egypt]* *Tel: +20 2 24567200* *https://www.novartis.com.eg/*

The recommended retention period for intracanal medication is no less than 14 days. However, recontamination of the canal may take place if the medicament is retained for 2 weeks ^29, 30^. That is why intracanal medicaments was placed for 72 hours in the present study.

*Second visit:* After 72 hours the patients returned and the intracanal medication was removed by irrigation with a sterile physiologic saline. Periapical radiograph was taken with the master gutta percha ProTaper cone *(Dentsply Maillefer, TN, USA)* for master cone verification. Obturation was done by modified single cone technique using ProTaper gutta percha points, ADseal resin sealer *(META BIOMED Co, Chungbuk, Korea #DM-15-4) and* auxiliaries gutta percha cones size 25 *(META BIOMED Co, Chungbuk, Korea).* A cotton pellet was placed in the pulp chamber and the access cavity was sealed with a temporary filling *.* Postoperative intraoral periapical radiograph was taken.

### Blinding

The study was double-blinded in which participants, the outcome assessor and data analyst were blinded in this trial. The operator couldn’t be blinded due to the nature of the study as the intracanal medicament must be exposed and can’t be masked.

Participants: Participants did not know which group they were treated with; Numbered papers indicating the intra-canal medicament to be used were packed in opaque closed envelopes by M.N., where the patient picked up an envelope. After mechanical preparation, operator M.O. opened the envelope and used the intra-canal medicament assigned to that patient according to the number present inside the envelope.

Outcome Assessor: The assistant supervisor (Y.N.) who collected the VAS, did not know which group the participants were assigned to.

Data Analyst: The assistant supervisor (N.N.) performed the statistical analysis.

### Harms

If any harm was seen in the participants either in intervention or control groups they were recorded and reported at the end of the trial. The treatment according to the harm:

- Pain: administration of Anti-inflammatory analgesics. (Brufen 600 mg, 1 tablet when needed).

- Swelling: hot fomentation, mouth rinse with salty warm water, antibiotic administration in-case of presence of fever or lymphadenopathy (Augmentin 1 gm, capsule every 12 hour for 5 days).

### Statistical analysis

Data were presented as means and standard deviations (SD) when appropriate. Data explored for normality was performed using D’Agostino-Pearson test. Independent t-test was used to compare between tested groups in age and mean pain (VAS). Chi square test was used to compare between tested groups for gender, teeth position and distribution and VAS scores. The significance level was set at P ≤ 0.05 (α=0.05). Intention to treat was used to adjust analysis for loss of patients during follow up period.

Statistical analysis was performed with IBM® SPSS® (IBM Corp. Released 2015. IBM SPSS Statistics for Windows, Version 23.0. Armonk, NY: IBM Corp.)

## Results

### I-Demographic data

93 patients were assessed for eligibility, 9 patients were excluded (5 were not meeting inclusion criteria while 4 declined to participate); in total 84 patients were randomly assigned, received intended treatment, and were analysed for the primary outcome (see underlying data ^21^). 3 participants were lost during follow up period after the first treatment session (2 from CH group and 1 from TAPC group) as they refused to continue and their treated tooth was extracted, implants to replace the extracted tooth were offered but none of the participants elected to have one. A CONSORT flow diagram is available as part of the reporting guidelines. Demographic data; age and gender had no significant difference between the two groups (P=0.966 and 0.141 respectively; Table 2 and Table 3).

**Table 2.** Means and standard deviations (SD) of age for different tested groups.

	Groups				p-value
	Group 1 (CH) ^1^		Group 2 (TAPC) ^2^		
	*Mean*	*SD*	*Mean*	*SD*	
**Age**	32.23	9.75	32.32	9.65	0.966 NS

NS=non-significant, *= Significant

*: Significant at P ≤ 0.05

*1. CH: Calcium hydroxide group.*

*2. TAPC: Triple antibiotic paste+catafast group*

**Table 3.** Frequency (N) and percentage (%) of gender for different tested groups.

		Groups				p-value
		Group 1 (CH) ^1^		Group 2 (TAPC) ^2^		
		*N*	*%*	*N*	*%*	
**Gender**	**Female**	14	35.0%	21	51.2%	0.141 NS
	**Male**	26	65.0%	20	48.8%	

NS=non-significant, *= Significant

*: Significant at P ≤ 0.05

*1. CH: Calcium hydroxide group.*

*2. TAPC: Triple antibiotic paste+catafast group.*

### II-Postoperative pain

**Figure 1.**
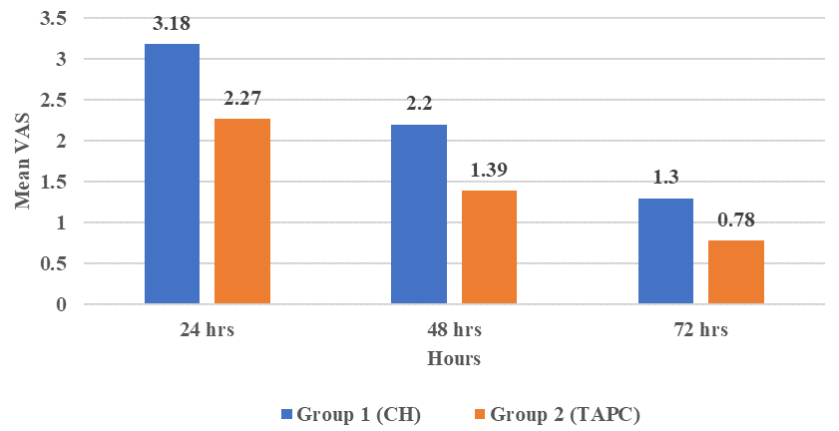
Bar chart showing mean visual analogue scale (VAS) for tested groups.

The overall highest incidence of pain values in both groups was at 24 hours postoperatively. Whereas the lowest incidence of pain values was at 72 hours postoperatively with a statistically significant difference between them (P≤0.001). The TAPC group showed statistically significant lower postoperative pain values at 48 hours only compared with the CH group (P=0.041), while at 24 and 72 hours there was no statistical significance (P=0.083 and 0.063, respectively) ( Figure 1 and Table 4).

**Table 4.** Means and standard deviations (SD) of visual analogue scale (VAS) for different tested groups.

		Groups				p-value
		Group 1 (CH) ^1^		Group 2 (TAPC) ^2^		
		*Mean*	*SD*	*Mean*	*SD*	
**VAS**	***24 hrs***	3.18 ^a^	2.37	2.27 ^a^	2.28	0.083 NS
	***48 hrs***	2.20 ^b^	2.00	1.39 ^b^	1.46	0.041 *
	***72 hrs***	1.30 ^c^	1.36	0.78 ^c^	1.11	0.063 NS
***p-value***		≤0.001 *		≤0.001 *		

*Different letters within each column indicates significant difference.*

NS=non-significant, *= Significant,

*: Significant at P ≤ 0.05.

*1. CH: Calcium hydroxide group.*

*2. TAPC: Triple antibiotic paste+catafast group.*

## Discussion

Post-endodontic pain is one of the major problems for both patients and dentists ^32^. Development of post-operative pain after root canal treatment frequently occurs. Several risk factors such as gender, age, type of intracanal medication used, presence of pre-operative pain, pulpal and periradicular diagnosis and apical extrusion of debris have been correlated with the occurrence of flare-ups ^33, 34^. Preoperative pain was recorded for each patient before starting treatment as it was considered a risk factor that can affect the postoperative pain ^35, 36^.

In this study, both groups were substantially homogeneous at diagnosis and had similar preoperative clinical conditions. Onay *et al. (2015)* ^34^ found that age, gender and tooth type did not influence the incidence of flare-ups. However, Arias *et al. (2013)* ^37^ and Sadaf *et al. (2014)* ^35^ reported that age, gender, tooth type and pre-operative pain are factors that influence post-operative pain.

In this study, factors affecting post-operative pain were excluded such as patients taking drugs that alter pain perception’ such as analgesics and antibiotics; patients with systemic diseases that could affect treatment; or patients suffering from psychological disturbances.

The purpose of this randomized clinical trial was to compare post-operative pain intensity after local medication with triple antibiotic paste together with an anti-inflammatory drug with that of calcium hydroxide. The incidence and intensity of postoperative pain was recorded at 24, 48 and 72 hours after placement of intra-canal medication.

An observable drop in pain levels in different follow up periods of both groups were recorded. This was in accordance to Pak *et al. (2011)* ^38^ who reported that post-treatment pain decreased substantially after 1–2 days of treatment and continued to drop to minimal levels after 7 days.

Although the TAPC group showed lower mean pain score levels at 24 and 72 hours than CH group, there was no statistically significant difference in the pain levels between both groups. While after 48 hours the mean pain score was significantly lower in TAPC group than CH with a statistically significant difference. Diclofenac has shown a substantial reduction of postendodontic pain when administered preoperatively in a single oral dose ^39^. Sharma *et al.* (1994) evaluated pain-relief post extraction in patients receiving oral ibuprofen. Pain-relief was attributed to the rapid absorption of granule formulation and/or a local action of ibuprofen in solution in the mouth ^40^. Therefore, potential advantages that may be acquired by the use of NSAIDs as intracanal medicament are: anti-inflammatory action and local analgesia.

Although calcium hydroxide is considered the gold standard for disinfecting root canals and has been widely used as an intra-canal medicament since 1920’s ^41^, its role in eliminating bacteria associated with apical infections is controversial. Microorganism have the ability to invade the dentinal tubules and buffer the high pH produced by calcium hydroxide compromising its antimicrobial activity ^42^. Calcium hydroxide release hydroxyl ions which increase alkalinity leading to death of bacteria and microorganisms inside the root canal ^42^.

Salem-Milani *et al.* was the first to compare anti-bacterial efficiency of ibuprofen, diclofenac and Ca (OH)2 using the agar diffusion test, they revealed the antibacterial properties of NSAIDs (ibuprofen, diclofenac) against E. faecalis; whereas Ca (OH)2 failed ^43^. The exact mechanism of antibacterial properties of diclofenac and ibuprofen remains unclarified. Studies have postulated the following mechanisms of action: inhibition of bacterial DNA synthesis, impairment of membrane activity, anti-plasmid activity, alteration in genes encoding transport/binding proteins, DNA synthesis and cell envelope as well as down-regulation of efflux pumps, reduced quorum sensing-controlled motility leading to reduced biofilm ^44^.

Additionally, some studies showed that calcium hydroxide is insufficient for the elimination of some symptoms, and thus, antibiotic pastes are used as an alternative ^45, 46^ due to their good antimicrobial and biocompatible properties ^47, 48^. This could be attributed to the combined spectrum of antimicrobial activity and synergetic or additive actions of antibiotics “ciprofloxacin, metronidazole, and minocycline” found in TAP. Individually, ciprofloxacin has a broad spectrum activity and acts against both Gram-positive and Gram-negative bacteria by inactivating enzymes and inhibiting cell division ^49^. Metronidazole is effective against obligate anaerobes, which are common in the deep dentin of infected root canals and acts by disrupting bacterial DNA ^49^. Minocycline is a broad-spectrum tetracycline antibiotic and acts by inhibiting protein synthesis and inhibiting matrix metalloproteinase enzyme ^49^. Combination of these three antibiotics overcomes bacterial resistance and achieves higher antimicrobial action ^18^. Previous studies have shown favorable results when antibiotic mixture of ciprofloxacin, metronidazole, and minocycline has been used as topical root canal agents ^18, 19, 50^. The absence of inter-appointment flare-up with TAP can also be attributed to the anti-inflammatory property of minocycline ^51^. Although antibiotic pastes have been successfully used in endodontic procedures, they should be completely removed before final root canal obturation to avoid their negative effects, such as tooth discoloration, cytotoxicity, and prevention of sealer or cement penetration into root dentin ^52, 53^. However, it is impossible to completely remove antibiotic pastes from the root canal using conventional irrigation protocol ^53– 55^.

This study is a randomized clinical trial conducted on a relatively large sample size male and female patients with age ranging between 18–50, in real clinical settings and was conducted efficiently so it may be reasonable to generalize the results. It proposes an alternative way for decreasing post-operative pain of asymptomatic uniradicular necrotic teeth.

Within the limitations of this study, it is recommended to use the TAPC intracanal medicaments in necrotic cases, which could lead to efficient pain relief post-operatively. It could also be recommended to modify the eligibility criteria to include teeth with irreversible pulpitis to evaluate the effect of the presence of inflamed vital pulpal tissues on the post-operative pain.

Further *in vivo* and immunological studies are needed to identify the exact mechanism by which the TAPC resulted in decreasing postoperative pain and to determine the cytotoxic effect of TAPC.

## Conclusion

Similar to calcium hydroxide, triple antibiotic paste with an anti-inflammatory drug is an excellent intracanal medicament for reducing post-operative pain in patients with uniradicular necrotic teeth.

## Data Availability

Figshare: The effect of triple antibiotic paste as an intracanal medication with an anti-inflammatory drug on post-operative pain of asymptomatic uniradicular necrotic teeth: a double blind randomized clinical trial. https://doi.org/10.6084/m9.figshare.8276981 ^21^ This project contains the following underlying data: Results.xlsx (Demographic and pain data for participants) Figshare: The effect of triple antibiotic paste as an intracanal medication with an anti-inflammatory drug on post-operative pain of asymptomatic uniradicular necrotic teeth: a double blind randomized clinical trial. https://doi.org/10.6084/m9.figshare.8276981 ^21^ This project contains the following extended data: DIAGNOSTIC CHART.docx (Form used to collect patient data) Pain scale chart english version.docx (Visual analogue pain scale, English) Pain scale chart arabic version.docx (Visual analogue pain scale, Arabic) Figshare: CONSORT checklist and flowchart for article ‘The effect of triple antibiotic paste as an intracanal medication with an anti-inflammatory drug on post-operative pain of asymptomatic uniradicular necrotic teeth: a double blind randomized clinical trial.’ https://doi.org/10.6084/m9.figshare.8276981 ^21^ Data are available under the terms of the Creative Commons Attribution 4.0 International License (CC BY 4.0)
