# Supplementary material for: The effect of triple antibiotic paste as an intracanal medicament with an anti-inflammatory agent on post-operative pain of asymptomatic uniradicular necrotic teeth: a double blind randomized clinical trial
Source: F1000Res. 2025 Dec 16;8:1687. Originally published 2019 Sep 25. [Version 3] doi: 10.12688/f1000research.19699.3 (PMC12811721; doi:10.12688/f1000research.19699.3)
Supplement: Peer review history

# Reviewer report: Reviewer response for version 3

**DOI:** 10.5256/f1000research.193658.r447921

**Published:** 2026-01-16

**Authors:** Engy Medhat Kataia

**Affiliations:** 1 The British University of Egypt, Cairo, Egypt

The work clearly presented and addresses a very important issue in endodontics.

 The most important factor for the success of a root canal treatment is disinfection; the two materials used in this study target the disinfection of the canal. Pain after endodontic treatment is common among patients, making them always feeling that the experience was unpleasant. the authors managed to add an anti-inflammatory drug to the tested material, which for sure helped decrease the postoperative pain and discomfort.

Is the work clearly and accurately presented and does it cite the current literature?

Yes

If applicable, is the statistical analysis and its interpretation appropriate?

Yes

Are all the source data underlying the results available to ensure full reproducibility?

Yes

Is the study design appropriate and is the work technically sound?

Yes

Are the conclusions drawn adequately supported by the results?

Yes

Are sufficient details of methods and analysis provided to allow replication by others?

Yes

Reviewer Expertise:

endodontics

I confirm that I have read this submission and believe that I have an appropriate level of expertise to confirm that it is of an acceptable scientific standard.

# Reviewer report: Reviewer response for version 3

**DOI:** 10.5256/f1000research.193658.r442135

**Published:** 2025-12-30

**Authors:** Maram E. Khallaf

**Affiliations:** 1 Restorative and Dental Materials Department, National Research Centre (NRC), Giza, Egypt

The manuscript presents a well designed and scientifically sound study that addressees an important research question.

 the required amendments are carefully done.

Is the work clearly and accurately presented and does it cite the current literature?

Partly

If applicable, is the statistical analysis and its interpretation appropriate?

Yes

Are all the source data underlying the results available to ensure full reproducibility?

Yes

Is the study design appropriate and is the work technically sound?

Yes

Are the conclusions drawn adequately supported by the results?

Yes

Are sufficient details of methods and analysis provided to allow replication by others?

Yes

Reviewer Expertise:

Endodontics,

I confirm that I have read this submission and believe that I have an appropriate level of expertise to confirm that it is of an acceptable scientific standard.

# Reviewer report: Reviewer response for version 2

**DOI:** 10.5256/f1000research.57963.r88089

**Published:** 2021-07-02

**Authors:** Jyothi Mandava, Ravichandra Ravi

**Affiliations:** 1 Department of Conservative Dentistry and Endodontics, Gandhi Institute of Technology and Management (GITAM) Dental College and Hospital, Visakhapatnam, India

**THIS ARTICLE IS AN ACCEPTABLE SCIENTIFIC STANDARD WITH SOME CORRECTIONS**

1. Title: Better to place "medicament" rather than medication and instead of drug agent.
  What is the meaning of uniradicular?
2. Need for the study and reasons for selecting these intracanal medicaments to test was not stated.
3. The objectives and hypothesis of the study were not discussed in the revised manuscript also.
4. In the discussion, it was reported that CH group has shown a significant correlation between postoperative pain with gender and age of patients but the statistical analysis was not provided (not included in the revised manuscript also).
5. Conclusions were not clear.
6. Participants: Preoperative pain was recorded-title was "Asymptomatic necrotic teeth".Then how can patients have preoperative pain?

Is the work clearly and accurately presented and does it cite the current literature?

Partly

If applicable, is the statistical analysis and its interpretation appropriate?

Yes

Are all the source data underlying the results available to ensure full reproducibility?

Partly

Is the study design appropriate and is the work technically sound?

Yes

Are the conclusions drawn adequately supported by the results?

Partly

Are sufficient details of methods and analysis provided to allow replication by others?

Yes

Reviewer Expertise:

Remineralization procedures in early carious lesions and in Regenarative procedures

We confirm that we have read this submission and believe that we have an appropriate level of expertise to confirm that it is of an acceptable scientific standard, however we have significant reservations, as outlined above.

# Reviewer report: Reviewer response for version 1

**DOI:** 10.5256/f1000research.21605.r66350

**Published:** 2020-08-10

**Authors:** Mahalaxmi Sekar, Kritika Selvakumar

**Affiliations:** 1 Department of Conservative Dentistry and Endodontics, SRM Dental College, Chennai, Tamil Nadu, India; 2 SRM Dental College, Ramapuram, Chennai, Tamil Nadu, India

This paper is aimed to comparatively evaluate the effectiveness of triple antibiotic paste with the anti-inflammatory drug diclofenac potassium and calcium hydroxide when used as an intra-canal medication in reducing post-operative pain.

**Introduction:**

 Should set the basis of the need for the study, with enough references to mitigate the study. 

1. Please state the lacunae in the current literature as to necessitate your study.
2. Mention the drawbacks of CH and the rationale of adding Catafast to TAPC. 
3. Mention the aim and objectives in the main text of the manuscript.
4. Further, the justification for choosing diclofenac over other anti-inflammatory drugs has not been highlighted.

**Methodology:**

 The article describes a novel double-blind randomized clinical trial and the author has clearly explained the process of selection of the subjects. Though the methodology has been elaborately explained, literature evidence reveals the use of TAP in the concentration of 1:1:1 as intracanal medicament, whereas in this study the concentration used is 500mg ciprofloxacin, 500mg metronidazole, 55mg minocycline, and 50mg diclofenac potassium. The low concentration ofdiclofenac used might not justify the role of an anti-inflammatory agent since it may negatively influence the results of the study. In addition, the authors have used commercial calcium hydroxide (metapex) comprising of iodoform which is an antiseptic agent that could also contribute to reducing post-operative pain.

**Results:**

 The pain was recorded at 24, 48, and 72 hours using VAS scale. During this period any participants suffering from pain or swelling both in the intervention and control groups were noted by the author as mentioned in the manuscript. However, the number of participants experiencing these symptoms and who received pharmacological therapy is not mentioned in the article, which might

 play a key role in influencing the overall results of the study. The author has analysed the age and gender comparison between the groups, however, the author could have subcategorised the wide age range of 18-50 years and denoted the number of participants falling under each category in the 2 groups, as it would be a major contributory factor.

**Discussion:**

 The intracanal medicaments are usually placed for about a week, the author could have discussed the reasons for choosing a short time interval of 24,48 and 72 hours. The author has substantiated with literature evidence on the use of ibuprofen for pain relief but has chosen diclofenac potassium. The author could have emphasized the use of diclofenac potassium with literature evidence, described the mechanism of action of the drug in improving the efficacy of TAP which would have added more value to the article. The author should have explained the purpose of using a lesser concentration of diclofenac potassium. The reasons for drawing the conclusions are also not clearly dealt with in the manuscript.

Is the work clearly and accurately presented and does it cite the current literature?

Partly

If applicable, is the statistical analysis and its interpretation appropriate?

I cannot comment. A qualified statistician is required.

Are all the source data underlying the results available to ensure full reproducibility?

No

Is the study design appropriate and is the work technically sound?

No

Are the conclusions drawn adequately supported by the results?

Partly

Are sufficient details of methods and analysis provided to allow replication by others?

Yes

Reviewer Expertise:

Endodontics, dentin bonding, regeneration, remineralisation

We confirm that we have read this submission and believe that we have an appropriate level of expertise to state that we do not consider it to be of an acceptable scientific standard, for reasons outlined above.

# Response

**Published:** 2021-06-18

**Authors:** Mohamed Omaia

**Affiliations:** October 6 university, Egypt

Drawbacks of CH and the rationale of adding Catafast to TAPC. had been mentioned in the discussion.

 While the mechanism of action of diclofenac potassium had been added to the new version of manuscript.

# Reviewer report: Reviewer response for version 1

**DOI:** 10.5256/f1000research.21605.r64970

**Published:** 2020-07-01

**Authors:** Jyothi Mandava, Ravichandra Ravi

**Affiliations:** 1 Department of Conservative Dentistry and Endodontics, Gandhi Institute of Technology and Management (GITAM) Dental College and Hospital, Visakhapatnam, India

This article is an acceptable scientific standard with some corrections:

1. The introduction part is not structured well and the flow of the written matter is missing.
2. The objectives and hypotheses of the study were not discussed.
3. In the discussion, it was reported that CH group has shown significant correlation between post-operative pain with gender and age of the patients but the statistical analysis was not provided.
4. Preferable to place definitive conclusions from the outcome of the study.

Is the work clearly and accurately presented and does it cite the current literature?

Partly

If applicable, is the statistical analysis and its interpretation appropriate?

Yes

Are all the source data underlying the results available to ensure full reproducibility?

Partly

Is the study design appropriate and is the work technically sound?

Yes

Are the conclusions drawn adequately supported by the results?

Partly

Are sufficient details of methods and analysis provided to allow replication by others?

Yes

Reviewer Expertise:

Remineralization procedures in early carious lesions and in Regenarative procedures

We confirm that we have read this submission and believe that we have an appropriate level of expertise to confirm that it is of an acceptable scientific standard.

# Reviewer report: Reviewer response for version 1

**DOI:** 10.5256/f1000research.21605.r55604

**Published:** 2019-11-18

**Authors:** Maram E. Khallaf

**Affiliations:** 1 Restorative and Dental Materials Department, National Research Centre (NRC), Giza, Egypt

Article addresses an important point during endodontic treatment which is post operative pain. A sound and clear clinical trial is done representing the true clinical situation.

 The use of triple antibiotic paste with the addition of an anti-inflammatory drug showed same results regarding the postoperative pain as calcium hydroxide.

 Results of the intracanal bacterial reduction are not clearly shown in the text or discussed. Article needs more recent citations.

Is the work clearly and accurately presented and does it cite the current literature?

Partly

If applicable, is the statistical analysis and its interpretation appropriate?

Yes

Are all the source data underlying the results available to ensure full reproducibility?

Yes

Is the study design appropriate and is the work technically sound?

Yes

Are the conclusions drawn adequately supported by the results?

Yes

Are sufficient details of methods and analysis provided to allow replication by others?

Yes

Reviewer Expertise:

Endodontics

I confirm that I have read this submission and believe that I have an appropriate level of expertise to confirm that it is of an acceptable scientific standard, however I have significant reservations, as outlined above.

# Response

**Published:** 2020-07-01

**Authors:** Mohamed Omaia

**Affiliations:** October 6 university, Egypt

Results of intracanal bacterial reduction as secondary outcome will be published in separate article.
